# Serum Catestatin Levels and Its Relationship with Disease Activity in Patients with Ankylosing Spondylitis

**DOI:** 10.5152/ArchRheumatol.2025.11014

**Published:** 2025-06-23

**Authors:** Tugba Alisik, Yagmur Cagla Reis Altan, Murat Alisik, Baris Nacir

**Affiliations:** 1 Department of Physical Medicine and Rehabilitation, Bolu Abant İzzet Baysal University, Bolu, Türkiye; 2 Department of Physical Medicine and Rehabilitation, Halil Şıvgın Çubuk State Hospital, Ankara, Türkiye; 3 Department of Medical Biochemistry, Bolu Abant İzzet Baysal University, Bolu, Türkiye; 4 Department of Physical Medicine and Rehabilitation, University of Health Sciences, Ankara Training and Research Hospital, Ankara, Türkiye

**Keywords:** Ankylosing spondylitis, catestatin, chromogranin A

## Abstract

**Background/Aims:**

Catestatin (CST) is a bioactive peptide with well-established cardiovascular roles, including anti-inflammatory and metabolic effects. This study aimed to compare the serum CST levels in patients with ankylosing spondylitis (AS) and healthy control subjects.

**Materials and Methods:**

This cross-sectional study included 95 patients with AS who met the Assessment of SpondyloArthritis International Society classification criteria for AS and 85 healthy individuals. Demographic, clinical, and laboratory parameters were recorded. Serum CST levels were measured by commercially available enzyme-linked immunosorbent assay kits.

**Results:**

Median serum CST levels were significantly lower in patients with AS compared to the control group (2.57 [1.74-4.58] vs 9.4 [3.55-26.6] ng/mL, *P* < .001). Catestatin levels were lower in the active AS group than in the inactive AS and control groups (*P* < .05). Significant negative correlations were found between CST and age, ankylosing spondylitis disease activity score–C-reactive protein (ASDAS-CRP), and C-reactive protein. Multiple linear regression analysis demonstrated that serum CST levels were significantly associated with the ASDAS-CRP group variable after model adjustment for sex, age, body mass index, HLA-B27 status, disease duration, and medications.

**Conclusion:**

These findings suggest that CST may play a role in the complex pathophysiology of AS. However, future multicenter longitudinal studies are necessary to further elucidate the relationship between CST and AS.

**Main Points**

- Serum catestatin levels are significantly lower in patients with ankylosing spondylitis compared to healthy controls.
- Lower catestatin levels are associated with active disease status in ankylosing spondylitis.

- The study highlights the potential role of catestatin in pathophysiology of ankylosing spondylitis.

## Introduction

Ankylosing spondylitis (AS) is an inflammatory rheumatic disease with unclear pathophysiology, characterized by chronic inflammation and pathological ossifications of axial joints and entheses, with a worldwide prevalence ranging from 0.1% to 0.5%.^1^ Involvement of the axial skeleton presents major clinical features, including limited spinal activity, inflammatory back pain, morning stiffness, and progression to ankyloses.^2^ Beyond physical impairment, AS can negatively affect the quality of life and contribute to psychological distress. Although cardiovascular involvement is considered a rare extra-articular manifestation, studies indicate that cardiovascular diseases are more common in AS patients than in the general population and represent the most common cause of mortality, with a rate of approximately 20%-40%.^3,4^ While the underlying mechanisms remain unclear, chronic inflammation is considered a fundamental contributor.^1^

Catestatin (CST) is a bioactive peptide derived from the proteolytic cleavage of chromogranin A, playing a crucial role in cardiovascular health.^5,6^ Catestatin inhibits the secretion and release of catecholamines by acting as a non-competitive inhibitor of neuronal nicotinic acetylcholine receptors.^7^ Additionally, CST suppresses macrophage activity, stimulates nitric oxide (NO) release, and exerts anti-inflammatory effects, contributing to its protective role in metabolic syndrome.^6,8^ Catestatin has been shown to regulate cytokine production and inhibit macrophage-driven atherosclerosis.^9^ Collectively, CST functions as a multifaceted peptide influencing blood pressure regulation, arrhythmias prevention, atherosclerosis mitigation, and anti-inflammatory responses.

Despite advances in understanding AS pathogenesis, specific biomarkers for disease diagnosis and monitoring remain lacking. Increased erythrocyte sedimentation rate (ESR) and increased C-reactive protein (CRP) serve as markers of chronic disease, yet their specificity is limited.^10^ To the best of the authors’ knowledge, there is no study evaluating the relationship between AS and CST.

This study aimed to compare serum CST levels between patients with AS and healthy controls and evaluate the relationship between CST levels and disease duration, disease activity, and inflammatory markers.

## Materials and Methods

This cross-sectional study was conducted between July 2023 and July 2024 and included 95 patients who applied to the Rheumatology Division of the Department of Physical Medicine and Rehabilitation of Ankara Training and Research Hospital with AS, meeting the criteria set forth by the Assessment of SpondyloArthritis International Society for AS. The control group consisted of 85 age- and sex-matched volunteers who applied for general health screening. These individuals had no known chronic or acute diseases, including cardiovascular, metabolic, autoimmune, or infectious conditions. They also had no symptoms, complaints, or relevant findings in their medical history. A comprehensive physical examination was conducted to confirm their health status, and those with any abnormalities were excluded. Additionally, individuals who were pregnant, breastfeeding, or using any medication were not included in the control group. All control subjects were evaluated for symptoms of systemic autoimmune or musculoskeletal inflammatory diseases, and laboratory tests, including complete blood count, differential blood count, and CRP levels, were performed to confirm the absence of inflammation, with those showing any abnormalities being excluded. Patients with chronic inflammatory disorders other than AS, those using lipid-lowering agents, or individuals with diabetes mellitus, renal impairment, heart failure, liver disease, bronchial asthma, chronic obstructive pulmonary disease, neoplasia, active infection, heavy alcohol consumption, or immune deficiency were excluded from the study. Prior to participation, all individuals were informed about the study procedure and provided written and verbal consent. This study was approved by Bolu Abant İzzet Baysal University Clinical Research Ethics Committee (Approval Number 2023/177; date: 06.06.2023) and followed the tenets of the Declaration of Helsinki.

Age, sex, body mass index (BMI), Bath Ankylosing Spondylitis Functional Index (BASFI), Ankylosing Spondylitis Disease Activity Score–C-Reactive Protein (ASDAS-CRP), disease duration (categorized into tertiles: 0-5 years, 5-10 years, and >10 years), treatment/medications, HLA-B27 positivity, and lipid profiles were noted. Blood samples were obtained after an overnight fasting state, kept for clotting, centrifuged for serum separation, and stored at −80 °C until the analysis. Triglyceride (TG), total cholesterol (TC), high-density lipoprotein cholesterol (HDL), and CRP levels were analyzed in an autoanalyzer (Architect c8000, Abbott Diagnostics, Chicago, IL, USA). The Friedewald formula was used to calculate low-density lipoprotein cholesterol (LDL) levels (LDL = TC-(HDL+(TG/5))). The levels of CST in the serum were quantified utilizing commercially available ELISA kits (BTlab, Shanghai Korain Biotech, Shanghai, China; catalog number: E4996Hu) following the operating procedure described by the manufacturer.

### Assessment of Disease Activity and Functional Status of Patients

Ankylosing spondylitis disease activity score–C-reactive protein was used to evaluate the disease activity of spondylarthritis. Ankylosing spondylitis disease activity score–C-reactive protein includes, in addition to the CRP value, the 4 self-reported items: duration of morning stiffness, peripheral pain/swelling, back pain, and overall assessment of the disease state by the patient.^11^ Bath ankylosing spondylitis functional index was employed to assess functional status. Bath ankylosing spondylitis functional index consists of 10 items that evaluate the impact of AS on daily activities, such as reaching, bending, and walking. Each item is scored from 0 (no difficulty) to 10 (impossible to perform), with the final score calculated as the mean of all items. Higher BASFI scores indicate greater functional impairment.^12^ The patients were divided into 2 groups: inactive (ASDAS-CRP < 2.1) and active AS (ASDAS-CRP ≥ 2.1).

### Statistical Analysis

A priori power analysis was conducted using data from the study by Zivkovic et al,^13^ with an effect size (Cohen’s d) of 0.56, an α error probability of 0.05, and a desired power of 0.95. The analysis indicated that at least 84 participants per group were required to achieve adequate statistical power.

Statistical analyses were performed using the SPSS software for Windows, version 21, released in 2012 (IBM SPSS Corp.; Armonk, NY, USA). The descriptive statistics were shown as mean ± SD for variables, median (1st-3rd quartile) for non-parametric distributions, or the number of cases (percentages (%)) for parametrically distributed, non-parametrically distributed, or categorical variables, respectively. The normality of distribution was determined by the Kolmogorov-Smirnov test and histograms. Pearson chi-square test was used for the comparison of categorical variables. Student’s *t*-test or one-way ANOVA test with post-hoc Bonferroni test was used to compare parametrically distributed variables. Other continuous variables were compared using the Mann-Whitney *U* test or the Kruskal-Wallis test with the Dunn-Bonferroni pairwise comparison test. Relationships between variables were evaluated by using Spearman’s correlation test. Multiple linear regression analysis was used to predict CST from predictors. *P* <.05 was used as the significance level.

## Results

There were no statistically significant differences in age, sex, and BMI between the AS patients and the control group (*P* > .05). The demographic, laboratory, and clinical characteristics are shown in Table 1. Among AS patients, 84 (88.4%) were HLA-B27 positive. Ankylosing spondylitis patients were categorized based on disease duration: 0-5 years (35.8%), 5-10 years (21.1%), and >10 years (43.2%). Median serum CST levels were significantly lower in patients with AS compared to controls (2.57 [1.74-4.58] vs 9.4 [3.55-26.6] ng/mL, *P* < .001).

Table 2 presents the comparisons of demographic, laboratory, and clinical characteristics among 48 patients in the active AS group, 47 patients in the inactive AS group, and 85 controls. Catestatin levels were significantly lower in the active AS group than in the inactive AS and control groups (*P* < .05 for both).

Significant negative correlations were found between CST levels and ASDAS-CRP, age, and CRP (Table 3). When comparing CST levels among different medication groups (None, Non-steroidal anti-inflammatory drugs [NSAID], Sulfasalazine [SLZ], anti-tumor necrosis factor [Anti-TNF], and NSAID+SLZ), no statistically significant difference was observed (*P* = .851).

In the multivariate linear regression analysis, the overall model did not show statistical significance (F(11, 83) = 1.494, *P* = .149), explaining 16.5% (R² = 0.165) of the variance in CST levels. Among the predictor variables, the ASDAS disease activity group (active vs. inactive) was the only statistically significant factor (*P* = .018), indicating that patients with high disease activity had significantly lower CST levels compared to those with low disease activity. Other variables, including sex, age, BMI, HLA-B27 status, disease duration, and medication use, were not significantly associated with CST levels (*P* > .05) (Table 4).

## Discussion

The study demonstrated that CST levels were significantly lower in patients with AS than in healthy control volunteers. Furthermore, CST levels in patients with active AS (ASDAS-CRP ≥ 2.1) were lower than in those with inactive AS. Additionally, CST levels showed significant negative correlations with age, ASDAS-CRP, and CRP levels. To the best of the authors’ knowledge, this is the first study to evaluate the serum CST levels in patients with AS.

Catestatin has been recognized for its immunomodulatory and metabolic regulatory functions in inflammatory diseases.^13^ Catestatin has been shown to exert anti-inflammatory effects by downregulating pro-inflammatory cytokines such as TNF-α and IL-6,^14^ both of which play critical roles in the pathogenesis of ankylosing spondylitis (AS).^15^ It was found that CST levels were significantly lower in AS patients compared to the control group and exhibited a negative correlation with the ASDAS-CRP, suggesting an inverse relationship between CST and disease activity. These results align with previous studies highlighting the relationship between CST and immune inflammation.^13,14,16^ The involvement of CST in these processes suggests it may influence both disease severity and progression by modulating inflammatory and metabolic pathways. Further studies are warranted to explore CST as a potential biomarker or therapeutic target in AS.

Several studies have shown that CST exerts antihypertensive, anti-inflammatory, antiarrhythmic, and cardioprotective effects.^5-9^ Chen et al^16^ found that lower CST levels were associated with increased coroner artery disease severity, and another study demonstrated a progressive decline in CST levels from stage A to stage C of heart failure.^17^ Catestatin also demonstrates antiatherogenic effects by influencing endothelial-leukocyte interactions via angiotensin-converting enzyme 2 (ACE2)-related mechanisms.^7^ Additionally, individuals with hypertension have been found to exhibit significantly lower plasma CST levels.^8^ The antihypertensive effects of CST include stimulating nitric oxide (NO) release and acting on receptors in the central nervous system.^8^ Moreover, in hypertensive patients, those with Type 2 Diabetes Mellitus have significantly decreased CST levels compared to non-diabetic individuals.^8^ Similarly, obese adolescents with metabolic syndrome (MS) exhibit lower serum CST concentrations compared to both obese adolescents without MS and the control group.^18^

Our findings suggest a potential connection between CST levels and AS. The lower CST levels observed in AS patients, particularly those with active disease, may reflect a dysregulated inflammatory and metabolic environment. However, CST levels have been reported to increase in certain conditions, such as advanced heart failure.^19^ In contrast, studies on coronary artery disease, heart failure, and metabolic syndrome have shown conflicting results.^9^ These discrepancies may result from the loss of CST’s inhibitory effect on catecholamine and chromogranin A secretion,^9^ differences in chromogranin A degradation mechanisms,^20,21^ or the generation of different CST variants from chromogranin A.^22^ Additionally, variations in the level of cardiac involvement among patients in the study samples may contribute to these differences.

It has been shown that CST levels are higher in inflammatory bowel disease (IBD), an inflammatory disease group.^13^ In a study, patients with Rheumatoid Arthritis (RA), a rheumatological disease with an increased risk of cardiovascular disease, have higher levels of CST than the control group.^5^ Moreover, disease activity score-28 levels were found to be positively correlated with CST levels.^5^ In contrast, this study found that lower CST levels were significantly associated with ASDAS-CRP, suggesting that AS may involve distinct inflammatory or neuroendocrine mechanisms compared to RA and IBD.

Research has shown that CST may play a role in modulating inflammation. The weak negative correlation between CST and disease activity indices (ASDAS-CRP) and CRP suggests that higher levels of CST may be associated with lower disease activity and inflammation in AS patients. This finding is consistent with the role of CST in modulating inflammatory responses,^18^ as lower disease activity often correlates with reduced inflammation.^23^ However, the correlation is weak, indicating that while CST may have some association with disease activity, it is not a robust predictor. Further research is needed to elucidate the mechanisms by which CST influences inflammation and explore its potential therapeutic implications in the management of AS.

C-reactive protein levels were significantly elevated in AS patients compared to controls, aligning with previous studies.^24,25^ A large-scale study of 851 AS patients reported elevated CRP levels in 61% of cases.^26^ Although the pathogenesis of AS is not fully known, increased CRP may function as a marker of chronic disease, but the need for a diagnostic marker remains due to its non-specificity.^10^ Additionally, a negative correlation between CST and CRP levels in AS was observed, suggesting a potential interplay between inflammatory processes and CST regulation. However, further studies are needed to elucidate the role of CST in AS and its association with inflammation.

Interestingly, no significant correlation between BASFI and CST levels was found. This finding suggests that CST, a known regulator of inflammation and cardiovascular function, may have a limited direct impact on functional impairment in patients with AS. The absence of a significant association could be attributed to the relatively small sample size or the complex interplay between inflammatory pathways and musculoskeletal function in AS.

The present study has several limitations. First, its cross-sectional design precludes the assessment of causal relationships. Second, the relatively small sample size may limit the generalizability of the findings. Third, chromogranin A levels and inflammatory parameters, such as interleukins and TNF-alpha, were not evaluated. Additionally, the study did not assess cardiac involvement or atherosclerotic risk in patients, which could provide further insights into systemic disease manifestations.

In conclusion, CST levels were lower in AS patients, particularly in active AS. These findings suggest that CST may play a role in the complex pathophysiology of AS. However, future multicenter longitudinal studies in which cardiac involvement of patients is assessed are necessary to better evaluate the relationship between AS and CST. Additionally, a significant negative correlation was observed between CST levels and both ASDAS-CRP and CRP, suggesting a potential relationship between CST, disease activity, and inflammation. Further research is needed to determine whether targeting CST pathways could support existing treatments or introduce new approaches to AS management.

## Figures and Tables

**Table 1.** Demographic, Laboratory, and Clinical Characteristics of the Ankylosing Spondylitis Group and the Control Group

Parameter	AS (N = 95)	Control (N = 85)	*P*
Age, years	41.78 ± 9.48	39.56 ± 9.59	.121*
Female, N (%)	35 (36.8%)	26 (30.6%)	.376**
BMI, kg/m^2^	25.8 (24.6-27.2)	25 (23.85-26.85)	.076^†^
N of positive HLAB27	84 (88.4%)		
Disease duration tertiles			
0-5 years	34 (35.8%)		
5-10 years	20 (21.1%)		
>10 years	41 (43.2%)		
Medication			
None	5 (5.3%)		
NSAID	39 (41.1%)		
SLZ	6 (6.3%)		
Anti-TNF	25 (26.3%)		
NSAID+SLZ	20 (21.1%)		
BASFI	1.87 ± 1.52		
ASDAS-CRP	2.07 ± 1.1		
ASDAS group			
Inactive AS	47 (49.5%)		
Active AS	48 (50.5%)		
Catestatin, ng/mL	2.57 (1.74-4.58)	9.4 (3.55-26.6)	<.001^†^
CRP, mg/L	4.7 (2.5-12)	1.7 (0.66-3.4)	<.001^†^
ESR, mm/hr	11 (4-17)	6 (4-9)	<.001^†^
Total cholesterol, mg/dL	185.24 ± 40.95	174.42 ± 46.56	.099*
Triglyceride, mg/dL	128 (91-193)	124.4 (89.55-166.2)	.463^†^
HDL, mg/dL	46 (38-55)	47 (41-54)	.281^†^
LDL, mg/dL	108.88 ± 34.6	110.5 ± 34.97	.755*

Data are expressed as the number (percentage), mean ± SD, or median (1st-3rd quartiles).

Anti-TNF, anti-tumor necrosis factor therapy; AS, ankylosing spondylitis; ASDAS-CRP, ankylosing spondylitis disease activity score–C-reactive protein; BASFI, bath ankylosing spondylitis functional index; BMI, body mass index; HDL, high density lipoprotein cholesterol; LDL, low-density lipoprotein cholesterol; NSAID, non-steroidal anti-inflammatory drug; SLZ, sulfasalazine.

*Student’s *t*-test.

**Chi-square test.

^†^Mann–Whitney *U* test.

**Table 2.** Demographic, Laboratory, and Clinical Characteristics of the Group

	Control (0)	Inactive (1)	Active (2)	*P*	1-0	2-0	2-1
Age, years	39.56 ± 9.59	39.32 ± 9.09	44.19 ± 9.33	.013*	>0.999	0.021	0.037
Female, N (%)	26 (30.6%)	19 (40.4%)	16 (33.3%)	.518**			
BMI, kg/m^2^	25.0 (23.9-26.9)	25.8 (24.9-27.4)	25.8 (24.4-27.0)	.199^†^			
BASFI		0.8 (0.3-1.6)	2.3 (1.2-3.6)	<.001^‡^			
ASDAS-CRP		1.4 (1-1.7)	2.8 (2.33-3.5)	<.001^‡^			
Disease duration tertiles				.044**			
0-5 years		18 (38.3%)	16 (33.3%)				
5-10 years		14 (29.8%)	6 (12.5%)				
>10 years		15 (31.9%)	26 (54.2%)				
Medication				.135**			
None		5 (10.6%)	0 (0%)				
NSAID		21 (44.7%)	18 (37.5%)				
SLZ		3 (6.4%)	3 (6.3%)				
Anti-TNF		10 (21.3%)	15 (31.3%)				
NSAID+SLZ		8 (17%)	12 (25%)				
Catestatin, ng/mL	9.4 (3.55-26.6)	2.84 (2.04-8.14)	2.23 (1.61-3.35)	<.001^†^	<0.001	<0.001	0.040
CRP, mg/L	1.7 (0.66-3.4)	3.6 (2.1-6.7)	8.35 (3.55-22.75)	<.001^†^	<0.001	<0.001	0.010
ESR, mm/hr	6 (4-9)	9 (4-16)	12 (5.25-18)	<.001^†^	0.064	<0.001	0.329
Total cholesterol, mg/dL	174.42 ± 46.56	179.51 ± 38.71	190.85 ± 42.69	.116*			
Triglyceride, mg/dL	124.4 (89.55-166.2)	144 (82-193)	123.5 (98-188.25)	.646^†^			
HDL, mg/dL	47 (41-54)	43 (40-58)	46.5 (36.25-52)	.503^†^			
LDL, mg/dL	110.5 ± 34.97	104.73 ± 33.11	112.94 ± 35.88	.493*			

Data are expressed as the number (percentage), mean ± SD or median (1st-3rd quartiles).

Anti-TNF, anti-tumor necrosis factor therapy; ASDAS-CRP, ankylosing spondylitis disease activity score–C-reactive protein; BASFI, bath ankylosing spondylitis functional index; BMI, body mass index; HDL, high density lipoprotein cholesterol; LDL, low-density lipoprotein cholesterol; NSAID, non-steroidal anti-inflammatory drug; SLZ, sulfasalazine.

*One-way ANOVA test.

**Chi-square test.

^†^Kruskal-Wallis test.

^‡^Mann-Whitney *U* test.

**Table 3.** Correlation Analysis Between Catestatin and Demographic, Laboratory, and Clinical Parameters

		Catestatin	ASDAS-CRP	BASFI
BASFI	rho	−0.140	0.585	
	*P*	.176	<.001	
	N	95	95	
ASDAS-CRP	rho	−0.303		
	*P*	.003		
	N	95		
Age	rho	−0.225	0.256	0.323
	*P*	.002	.012	.001
	N	180	95	95
CRP	rho	−0.371	0.489	0.059
	*P*	<.001	<.001	.569
	N	180	95	95
BMI	rho	0.006	−0.049	0.078
	*P*	.933	.636	.452
	N	180	95	95
ESR	rho	−0.322	0.194	0.054
	*P*	<.001	.059	.605
	N	180	95	95
Total cholesterol	rho	−0.177	0.178	0.179
	*P*	.018	.084	.083
	N	180	95	95
Triglyceride	rho	−0.136	−0.043	0.081
	*P*	.068	.678	.433
	N	180	95	95
HDL	rho	0.075	−0.029	−0.127
	*P*	.314	.779	.219
	N	180	95	95
LDL	rho	−0.018	0.126	0.160
	*P*	.815	.223	.121
	N	180	95	95

ASDAS-CRP, ankylosing spondylitis disease activity score–C-reactive protein; BASDAI, bath ankylosing spondylitis disease activity index; BASFI, bath ankylosing spondylitis functional index; BMI, body mass index; HDL, high density lipoprotein cholesterol; LDL, low-density lipoprotein cholesterol.

**Table 4.** Multivariate Linear Regression Analysis of Factors Associated with Catestatin Levels

Parameter	β ± SE	95% CI for β	*P*
Sex			
Male vs. female	−0.381 ± 2.348	−0.037 (−0.489 to 0.415)	.871
Age	−0.220 ± 0.139	−0.201 (−0.454 to 0.052)	.117
BMI	0.712 ± 0.428	0.175 (−0.034 to 0.384)	.100
HLA-B27			
Negative vs. positive	0.512 ± 3.392	0.050 (−0.603 to 0.702)	.880
Disease duration			
5-10 years vs. 0-5 years	−3.528 ± 3.085	−0.341 (−0.935 to 0.252)	.256
>10 years vs. 0-5 years	3.167 ± 3.125	0.306 (−0.295 to 0.908)	.314
Medication use			
NSAIDs vs. none	−1.853 ± 5.104	−0.179 (−1.161 to 0.803)	.717
Sulfasalazine vs. none	−1.818 ± 6.438	−0.176 (−1.414 to 1.063)	.778
Anti-TNF vs. none	0.129 ± 5.437	0.013 (−1.034 to 1.059)	.981
NSAIDs + SLZ vs. none	2.796 ± 5.582	0.271 (−0.803 to 1.344)	.618
ASDAS group			
Active vs. Inactive	−5.441 ± 2.261	−0.526 (−0.961 to −0.091)	**.018**

Statistically significant *P* values (*P* < .05) are shown in bold. Anti-TNF, anti-tumor necrosis factor therapy; ASDAS, ankylosing spondylitis disease activity score; CI, confidence interval; NSAID, non-steroidal anti-inflammatory drug; SE, standard error; SLZ, sulfasalazine; β, regression coefficient.

## Data Availability

The data that support the findings of this study are available from the corresponding author upon reasonable request.
